# Novel method to determine recursive filtration and noise reduction in fluoroscopic imaging – a comparison of four different vendors

**DOI:** 10.1002/acm2.13115

**Published:** 2020-12-14

**Authors:** Bente Konst, Jacob Nøtthellen, Stine Nalum Naess, Magnus Båth

**Affiliations:** ^1^ Department of Radiology Vestfold Hospital Trust Tønsberg Norway; ^2^ Faculty of Mathematics and Natural Sciences Department of Physics University of Oslo Oslo Norway; ^3^ Oslo University Hospital Oslo Norway; ^4^ Department of Medical Physics and Biomedical Engineering Sahlgrenska University Hospital Gothenburg Sweden; ^5^ Department of Radiation Physics Institute of Clinical Sciences Sahlgrenska Academy at University of Gothenburg Gothenburg Sweden

**Keywords:** fluoroscopy, noise, optimization, quality assurance, quality control, recursive filtration

## Abstract

**Purpose:**

This study attempted to develop a method to measure the applied recursive filtration and to determine the noise reduction of four different fluoroscopic systems. The study also attempted to elucidate the importance of considering the recursive filter for quality control tests concerning signal‐to‐noise ratio (SNR) or image quality. The vendor’s settings for recursive filtration factor (β) are, unfortunately, often not available. Hence, a method to determine the recursive filtration and associated noise reduction would be useful.

**Method:**

The recursive filter was determined by using a single fluoroscopic series and the method presented in this study. The theoretical noise reduction based on the choice of β was presented. In addition, the corresponding noise reduction, evaluated as the ratio of the standard deviation of the pixel value between a series with β equal to zero (recursive filtration off) and β > 0, was determined for different pulse rates given by pulses per second (pps), doses (mAs) and recursive filter. The images were acquired using clinically relevant radiation quality and quantity.

**Results:**

The presented method to measure the recursive filter exhibited high accuracy (1.08%) and precision (1.48%). The recursive filtration and noise reduction were measured for several settings for each vendor. The recursive filtration settings and associated recursive filtration factors for four different vendors were presented.

**Conclusions:**

This study presented an accurate method to determine applied recursive filtration, which was easy to determine. Hence, for all quality control purposes, including noise evaluation, it was possible to consider the essential noise reduction given by the settings for recursive filtration. It was also possible to compare the recursive filtration settings and associated recursive filtration within and between vendors.

## 1 Introduction

The main function of the fluoroscope is to provide real‐time dynamic images of moving internal structures and fluids in interventional radiology and as guidance for surgery. Real‐time computerized fluoroscopy was developed at the University of Wisconsin in the mid‐1970s.^1^ During the last several decades, there has been notable development in detector design and rapid advances in the possibilities for postprocessing software.

As early as 1973, lag was described as a difficulty with the images obtained from angiographic procedures.^2^ Lag denotes the phenomenon in which information from previous image frames persists in the current frame.^3^, ^4^ An effect of image lag is blurring of moving objects in the radiation field, which may result in image smearing or persistence, comet artifacts and streak artifacts.^3^, ^5^ Lag|_1_ denotes the ratio of the signal in the first frame and frame 0, which is the frame subsequent to a radiographic exposure. The image lag for flat‐panel detectors has been reported to be 2%–10% for an a‐Si detector depending on incident exposure,^6^ 0.4%–1.5% and 4%–5% for a‐Se detectors,^6^, ^7^ and less than 0.1%^8^ for CMOS detectors. Image intensifiers coupled to analogous cameras may have as much as 20% lag.^9^ The lag degrades the temporal resolution of the dynamic image. However, lag also increases quality in images where there is no motion because images are integrated, which again reduces the quantum noise (averaging uncorrelated noise).

Lag due to inherent detector properties is dependent on pulse rate (integration time) and may reduce the noise power spectrum (NPS) by 15%.^10^ If image lag is not corrected, lag will reduce the NPS, and the determined detective quantum efficiency (DQE) will increase.^10^, ^11^, ^12^, ^13^ Fluoroscopic systems with lag will erroneously be assessed to have higher DQE compared to systems with less lag. The determined DQE can be 15%–40% higher than the DQE without lag. In addition, it is needed to determine the temporal modulation transfer function (MTF).^13^

Lag was intentionally introduced by recursive filtration in the beginning of 1980s to reduce noise by using information from previous images in a new image as a temporal averaging of image signals.^1^, ^14^, ^15^ This technique may also be called recursive integrating, weighted frame averaging, fluoroscopic noise reduction (FNR) and temporal frequency filtering. A β‐factor describes weighting in a simple temporal recursive filtering method, extensively used in fluoroscopy.^9^ Recursive filtering is often user adjustable and has effects on both the correlated and the noncorrelated part of the NPS. Hence, SNR measurements in fluoroscopic imaging have to compensate for the β‐factor. Both the noise reduction and lag increase with decreasing β. Therefore, it is necessary to use an appropriate noise reduction factor according to the amount of motion in the image.^16^

Software and hardware development has increased the possibility for digital image processing. The pure recursive filter might be replaced by adaptive temporal filtering or real‐time image noise reduction technology (INRT). To determine regions that contain moving structures, INRT reduces image lag by adjusting the filter weight depending on the correlation of pixels in successive frames. The noise reduction can be both temporal and spatial. Temporal refers to subsequent images, and spatial refers to a process carried out over an area within an image (Philips Healthcare 2012). In terms of noise reduction, any integration of images is comparable to an increase in dose.

Quality control tests are often performed using a phantom, and exposure settings, such as kV, mAs, pps and tube filtration, are usually described.^17^ It is notably common to evaluate fluoroscopic systems without considering the recursive filtering effects on the images.^18^, ^19^, ^20^ For quality control of an X‐ray system, it is important to differentiate between the performance of the system and the user’s ability to use the optimized system. The noise reduction due to temporal recursive filtration (preprocessing) is achieved at the cost of lag. This study focuses on the noise reduction due to recursive filtration. First, a detailed theory behind the basic recursive filtration is given. Then, a novel method to determine the recursive filtration is presented. Different vendors have different parameters that represent the β*‐*factor; thus, it may be difficult to compare vendors without performing a measurement of the β‐factor. This research demonstrates the importance of considering the noise reduction due to recursive filter when performing quality control measurements using a static phantom.

## 2 Theory

### 2.1 Recursive algorithm

Recursive filtering is applied to reduce noise by averaging frames. In terms of noise, simple averaging of four frames is comparable to increasing the dose by a factor of 4 and will reduce the quantum mottle by a factor of 2. A frame is defined as the signal from one pulse, whereas an image may be a result of averaging several frames. Recursive filtering is defined as a weighted sum of the current frame with the previous image based on several frames. This filtering is a digital filter described as an infinite impulse response filter (IIR), where the displayed image *I(n)* is given by

(1) I(n)=βS(n)+(1‐β)I(n‐1)

where n denotes the frame number, *S(n)* is the measured signal of frame *n*, and β is a value from 0 to 1.^14^, ^21^, ^22^ Recursive temporal filtering adds a fraction of previous frames to the current frame. *I(n)*, the effective number of photons used to create the currently viewed image is increased, and the variance is reduced. The smaller the numerical value of β is, the greater the weight from previous frames and the noise reduction is; thus, there is a greater potential for lag. The parameter β is sometimes referred to as the “forget factor”. The closer β is to one, the more quickly the filter forgets the old input.

In general, for a weighted sum of frames generated from pulses of *Q* photons, the displayed image is

(2) $$I\left(n\right)=\sum_{i=0}^{N‐1}\alpha_{i}\gamma Q_{i}$$

where the sum of coefficients *α_i_* equals 1, *i* is the frame index (*i* = 0 is the last frame), the γ is a value generated from a lookup table describing the conversion from *Q* to the measured signal of a frame, and *N* is the total number of frames. For images with recursive filtering, β is a function of the weight factor *α_i_*, the pulse order *i* and the number of averaged frames, *N*. The displayed image using recursive filter is given by

(3) $$I\left(n\right)=\beta \sum_{i=0}^{N‐1}{\left(1‐\beta \right)}^{i}S_{i}$$

where *S_i_* = γ*Q_i_* ^23^, ^24^.

Using basic properties for variance (Var($\sum_{i=0}^{N}X_{i})=\sum_{i=0}^{N}Var\left(X_{i}\right)$ and Var(aX)=a^2^Var(X)), the noise (standard deviation) is given by

(4) $$\sigma_{with\;recursive}=\sqrt{\sum_{i=0}^{N‐1}\alpha_{i}^{2}Var(S_{i}})$$

By comparing Eqs (2) and (3), it is obvious that *α_i_* in Eq (2) is equal to β*(1‐*β*)^i^* and depends on β and the number of averaged frames (*N*). Then, the noise in the image is given by

(5) $$\sigma_{with\;recursive}=\sqrt{\sum_{i=0}^{N‐1}{\left(\beta \cdot {\left(1‐\beta \right)}^{i}\right)}^{2}Var\left(S_{i}\right)}=\sqrt{\frac{\beta ({\left(1‐\beta \right)}^{2N}‐1)}{\beta ‐2}Var\left(S\right)}$$

where it is assumed the variance of each frame S_i_ is constant and equal to Var(S), and the sum is calculated using a free version of Wolfram Alpha.^25^ Averaging a sufficient number of frames according to the value of β results in a noise in the displayed image that (Eq 5) approaches^26^:

(6) $$\sigma_{with\;recursive}=\sqrt{\frac{‐\beta }{\beta ‐2}Var(S})=\sqrt{\frac{\beta }{2‐\beta }}\cdot \sigma_{\sin gle\;frame}$$

where

(7) $$\sigma_{\sin gle\;frame}=\sigma_{without\;recursive}=\sqrt{Var(S})$$

The number of frames required to approach Eq (6) increases for lower values of β. The β derived here does not distinguish between noise reduction due to detector lag and the recursive filter.

The ratio between noise^2^ with and without recursive filter is given by

(8) $$R=\frac{\sigma_{\mathrm{with}}^{2}}{\sigma_{\mathrm{without}}^{2}}=\frac{\beta }{2‐\beta }$$

By using recursive filtration, the pixel value in the first images will, in practice, be reduced, depending on the choice of weighting factor β. Thus, the recursive filtration is not used for the first images, or the first images are not shown. Different vendors have different solutions for the very first images in a fluoroscopic series using recursive filtration.

The reduction in noise given by Eq (6) is similar to a dose increase given by 1/noise^2^; hence, the corresponding dose increase is given by

(9) $$C\text{orresponding}\;dose\;increase\;=\frac{1}{\sigma_{\mathrm{reduction}}^{2}}=\frac{2‐\beta }{\beta }=N_{\mathrm{eff}}.$$

*N_eff_* is the effective number of frames that have to be averaged to achieve the same noise level, but at the expense of higher lag if recursive filtration is omitted. The relative expected noise reduction is given by Eq (10).

(10) $$\text{Relative noise reduction}=\frac{\sigma_{\mathrm{without}}‐\sigma_{\mathrm{with}}}{\sigma_{\mathrm{without}}}=1‐\sqrt{R}$$

**Fig. 1:**
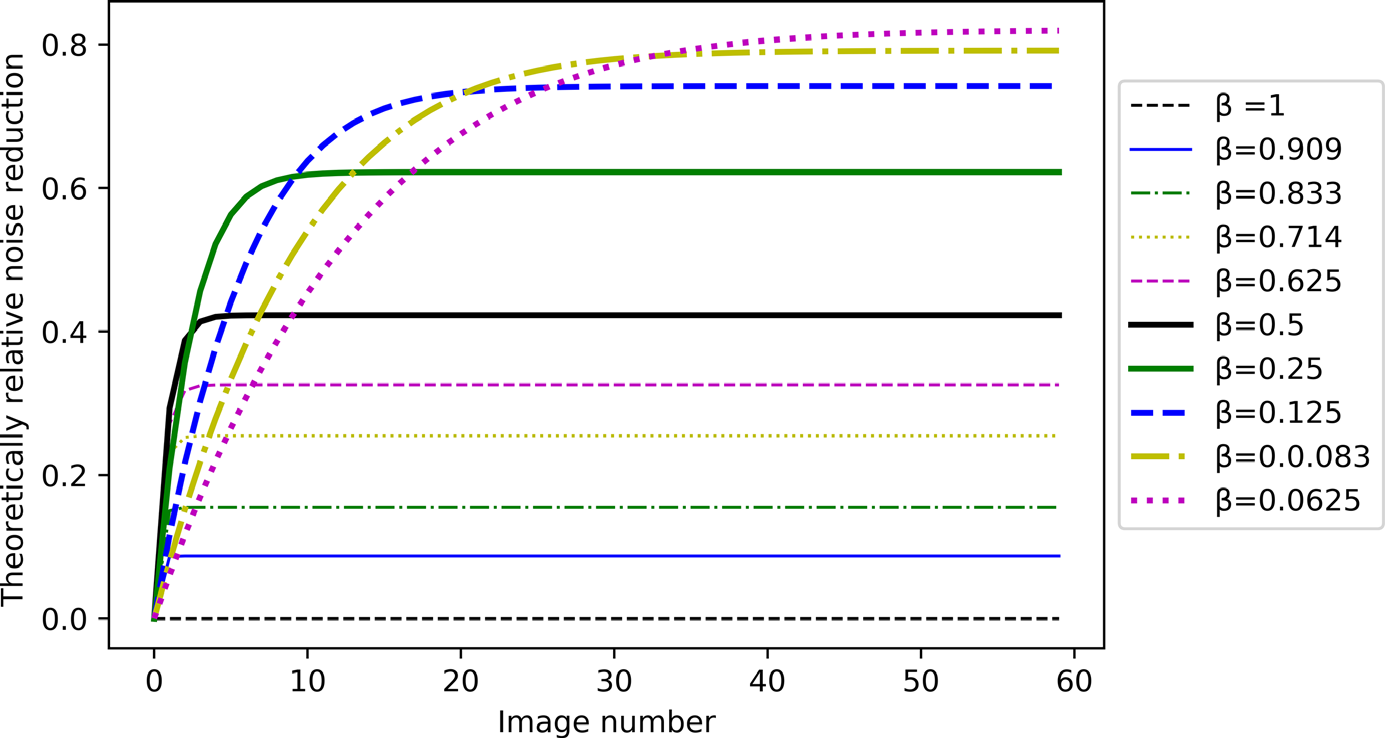
Theoretical noise reduction (noise using recursive filtration compared to not using recursive filtration (*β* = 1)) as a function of image number and applied recursive filtration (*β*).

The theoretical noise reduction as a function of image number for different values of β factor is shown in Fig. 1.

If β is unknown, it can in theory be determined by comparing an image with and without recursive filtration and calculating the ratio, R. β is then given by Eq (11).

(11) $$\beta =\frac{2R}{R+1}$$

When using Eq (11), some precautions must be taken to assure that R is determined under equal conditions. In practice, some X‐ray systems have look‐up tables with different shapes and slopes to create contrast. They might even be adaptive, which means that each pixel or area of pixels have dedicated look‐up tables. A novel and applicable method to determine *β* using three images from one fluoroscopic series and their associated variances is outlined below.

### 2.2 Determination of β using variances of homogenous images

All images within the fluoroscopic series are correlated similarly due to standard image processing. In addition, they contain uncorrelated Poisson noise and fixed pattern noise. The images are correlated, but the pulses are uncorrelated or independent. Using recursive filtering, the neighboring images are correlated due to recursive filtration, but images with sufficient time separation do not have such correlation.^27^ It is assumed that the varying noise is equal for added and subtracted images when recursive filtration is off since there is no correlated noise. Using recursive filtration, the variable noise will be less in subtracted neighboring images than in added neighboring images due to the correlation.

Using three images from one fluoroscopic series, the last two images in the series (neighboring images and not the last hold image), and an image in the middle of the series, it is possible to determine β using variances of added (suffix imadd) and subtracted images (suffix imdiff) of neighboring images (suffix n) and images with some distance (suffix d).

From the variance of added and subtracted images, acquired sufficiently separated not to be correlated due to lag, it is possible to separate the variance from correlated fixed pattern noise (suffix fp) according to Eq (12) ^28^:

(12) $$\sigma_{\mathrm{fp}}^{2}=\sigma_{imadd,\;d}^{2}‐\sigma_{imdiff,\;d}^{2}$$

The variance of the added neighboring image in a fluoroscopic series contains multiplicative varying nonstochastic noise (e.g., image processing, pixel size), stochastic noise (correlated and uncorrelated quantum noise), and fixed pattern noise. Using Eq (12), the variance of added neighboring images can be corrected for correlated fixed pattern noise as shown in Eq (13):

(13) $$\sigma_{imadd,\;n,\;corr}^{2}=\sigma_{imadd,\;n}^{2}‐\sigma_{\mathrm{fp}}^{2}$$

Hence, the imadd, n, corr contains multiplicative varying nonstochastic noise, uncorrelated stochastic noise (quantum noise), and correlated noise due to the recursive filter, but no correlated fixed pattern noise. The variance of *subtracted* images in a fluoroscopic series contains multiplicative varying nonstochastic noise, uncorrelated stochastic noise, and *reduced* correlated stochastic noise due to the recursive filter. Thus, the ratio between images with and without recursive filtration can be written as given in Eq (14):

(14) $$R={\left(\frac{\sigma_{\mathrm{with}}}{\sigma_{\mathrm{without}}}\right)}^{2}={\left(\frac{\sigma_{imdiff,n}}{\sigma_{imadd,n,corr}}\right)}^{2}$$

Alternative recursive algorithms can be used by replacing β with, for example, 1/*K,* where *K* = 1, 2, 4, 8, or 16. This algorithm, or a close relation to it, is in use by several fluoroscopic C‐arm units,^4^ and this K‐factor is used further in our study. Even if the K‐factor is an integer, it does not imply that an integer number of images is averaged,the images are weighted. The C‐arm systems may have different noise reduction for last image hold and for the live images. Some systems also have automatic detection of motion. If substantial motion is present in the image, the minimum noise reduction is applied, some for the whole image. A more sophisticated system also reduces noise in areas of the image where no motion is present. This will affect the perceived noise and lag in the system. All vendors have introduced a type of recursive filtering/noise reduction by using the signal from the very same photons repeatedly in an image. The guidelines for optimal choices of weighting of old versus new frames and number of frames averaged in the displayed image are usually insufficient in the vendor’s user manual. In addition, lack of definition of the vendor’s noise reduction factor makes it difficult to compare system settings. This study aims to present a method to determine the applied noise reduction factor K and to reveal the relationship between noise reduction and applied settings for recursive filtration for four different vendors.

## 3 Method

### 3.1 Description of C‐arms

C‐arms from four different vendors were evaluated in this study: GE Healthcare (OEC Elite) Philips Medical Systems (Zenition), Siemens Medical Systems (Cios Alpha), and Ziehm (Vison RFD Hybrid Edition). They were all equipped with flat panel X‐ray detectors, but with different sensor designs. See Table 1 for specifications for the actual C‐arms.

**Table 1:** Description of the X‐ray systems (( ^29^), ( ^30^), ( ^31^) and ( ^32^))

Manufacturer, model/date	GE OEC Elite 2019	Philips Zenition 70 2019	Siemens Cios Alpha 2017	Ziehm Vision RFD Hybrid edition 2018
Detector type	GE CMOS	Trixell Amorphous Silicon	Varian PaxScan 3030X	Dexela CMOS
Scintillator	Cesium iodide	Cesium iodide	Cesium iodide	Cesium iodide
Pixel pitch or size (µm)	198	154	194	100
Active detector area (cm × cm) (pixel × pixel)	30.7 × 30.2 1548 × 1524	20.7 × 20.7 1344 × 1344	29.8 × 29.8 1536 × 1536	30.7 × 30.7 3072 × 3072
FOV 0, Normal (cm)	31 × 31	20.7 × 20.7	30 × 30	30.7 × 30.7
FOV 1 or MAG1 (cm)	21 × 21	15.4 × 15.4	20 × 20	20.5 × 20.5
FOV 2 or MAG 2 (cm)	15 × 15	11.0 × 11.0	15 × 15	15.4 × 15.4
Software version (DICOM tag 0018,1020)	1.0.4520	PH Mobile C R5.1	VG20X\VA20D	7.06.2.23 VCSID: ab5474d Date: 2019‐05‐06 Build: 23

For C‐arms, there are often restrictions on the choices of exposure parameters, that is, it may not be possible to change kV and mAs separately. Therefore, a normal patient was simulated using a 20‐cm PMMA slab, and the exposure parameters were given by the automatic exposure control. Hence, the images were acquired using clinically relevant techniques. Series were acquired using different levels of recursive filtration. Some vendors offer different kinds of noise reduction.

For the GE system, it is not possible to change the recursive parameters beyond min, low, medium and high. For Philips, the user interface allows choosing between activating a “reduce noise” or “reduce blur” button. In the default setting, none of them are activated. To change the temporal noise reduction taste (TNRT) values allocated for the buttons, a service key and password are needed.

From the user interface of the Siemens C‐arm, it was possible to change the preprogramed K‐factor by pressing an integration button low or high, which change the K‐factor by 0.7 and 1.4, respectively. By using a service password for the Siemens system, it was possible to choose a desired recursive filtration independent of the applied pps, dose, and FOV. In addition, it was possible to choose whether motion correction should be used. For Ziehm, the user can choose recursive filtration off or level 1, 2, 3, or Auto. By using a service password, it is possible to change the recursive filtration allocated to the buttons 1, 2, and 3. A service key enables turning on and off Ziehm Adaptive Image Processing (ZAIP) and even changing parameters for spatial and temporal strength and domain. See Table 2 for more details regarding settings for recursive filtration.

**Table 2:** Summary of possible recursive values for each vendor

Manufacturer/Model	Recursive filter adjustable in protocol set up using either password or service key.	Noise suppression — adjustable in the daily user interface.
GE OEC Elite CFD	None	4 levels: minimum, low, medium, high
Philips Zenition 70	TRNT 00‐19	3 levels: Temporal reduce blur (button pressed) Temporal noise reduction (no button pressed) Temporal reduce noise (button pressed)
Siemens Cios Alpha	K1:8 Number of images averaged for LIH. M, threshold for motion detection	Integration factor low (0.7), medium (1), high (1.4)
Ziehm Vison R FD Hybrid Edition	1:16 As standard ZAIP is always activated	Off, 1, 2, 3

### 3.2 Parameter settings

The image series were acquired using automatic exposure control and the protocol/exposure mode, recursive filtration, pulses per second, and field of view, as given in Table 3.

**Table 3:** Parameters applied for acquiring image series for determination of recursive filtration

System	Recursive filter	PPS	FOV	Protocol/exposure mode
GE	Min, medium, low high	4,8,15	FOV 0 FOV 2	General HD: Fluoro, HLF and cine
Philips	TNRT: 0‐19	4,8,15		Uro, Bladder
Siemens	OFF; K2, 1, M0; K4, 2, M0; K8, 2 M 0, and with weight factor. M0 denote no motion correction	1,3,7.5	FOV 0 FOV 2 FOV 3	Uro Standard Low, normal, and high dose
Ziehm	Off, 2‐16 with and without ZAIP	4,8, 12.5	FOV 0	Uro

### 3.3 Determination of recursive filtration and noise reduction

A homogenous phantom of PMMA, 30 cm × 30 cm, and thickness 20 cm was imaged with an air gap of 6 cm from the detector. For each C‐arm, 20 equal series were recorded for one exposure setting to study the variations in the measurements. In addition, three equal series were acquired for each setting, and the results of K‐factor and noise reduction were averaged.

The series lasted for 8–10 s, and all images were saved to a memory stick.

**Fig. 2:**
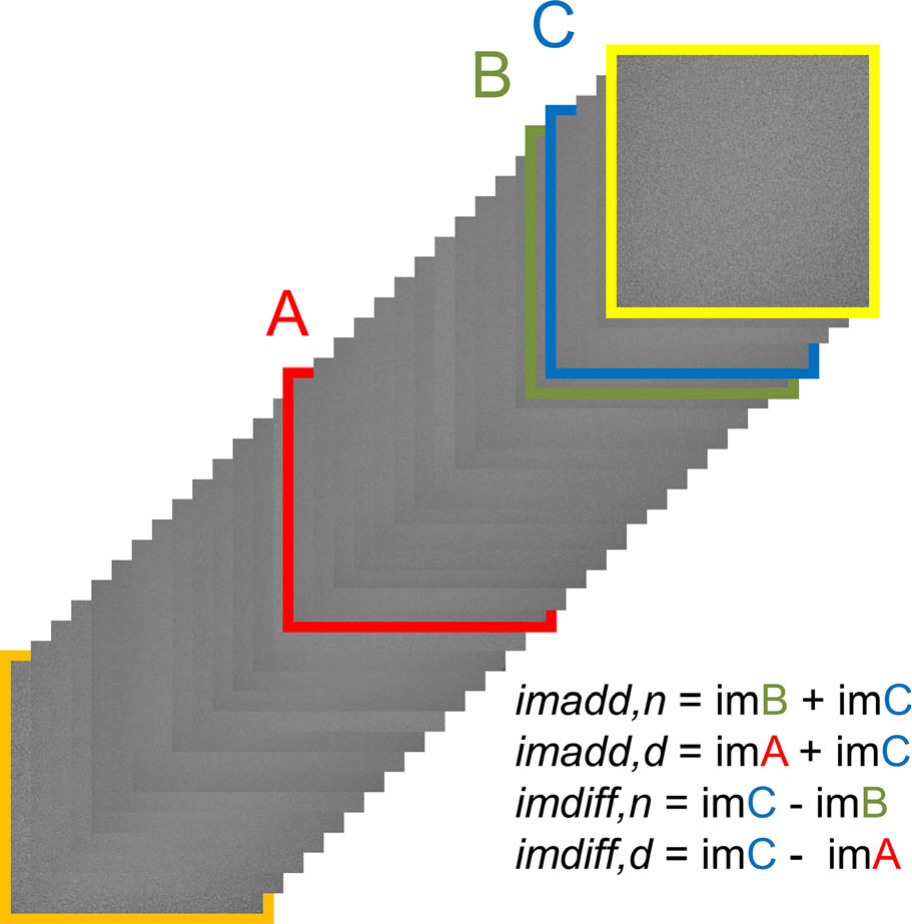
Example of an image series. The images with red, green, and blue frames were used to calculate the K‐factor. The image with the orange frame is the first image displayed by the X‐ray system and is the image with the most noise in the series. The image with the yellow frame is the last image in the stack, also denoted last image hold (LIH). LIH may have another K‐factor than the series and is therefore left out of the calculations

To ensure equal conditions, this method to calculate the K‐factor uses images from one fluoroscopic series instead of two. Three images were selected from the series of homogenous images, one in the middle of the stack, and the fourth and fifth to the last image, which is illustrated as the images with red, green, and blue frames in Fig. 2. From these three images, four new images are calculated, as shown in Fig. 2, and as described in section 2.2. Then, the variances of the four new images are calculated. By substitution of Eqs. (12) and (13) into Eq (14), the ratio between images with and without recursive filtration was calculated using Eq 15 and the four variances:

(15) $$R=\frac{\sigma_{imdiff,n}^{2}}{\sigma_{imadd,n}^{2}‐\sigma_{imadd,d}^{2}+\sigma_{imdiff,d}^{2}}$$

Finally, by substitution of *β* = 1/*K* into Eq (11), the K‐factor is determined using Eq (16):

(16) $$K=\frac{R+1}{2R}$$

The expected relative noise reduction was calculated using the K‐factor determined from one image series, that is, by substituting R from Eqs. (15) into Eq (10). The relative noise reduction due to recursive filter was also determined using two series of homogenous images. One series acquired with recursive filter off and one with recursive filter, and their associated variances according to Eq (10). All calculations and image processing were performed using Python 3.7.

## 4 Results

### 4.1 Measured recursive filtration

The accuracies of the measured recursive filtration based on 20 equal image series, given as the deviation from the chosen value on the C‐arm (nominal value), were 0.31% and 1.08% for Ziehm and Siemens, respectively, see Table 4. Associated precision rates, given as the coefficient of variation (CV), were 1.48% and 0.65%. The nominal values were not accessible for two vendors: GE due to information classified as proprietary, and Philips due to a change in the notation from K = 0–19 to TNRT values from 0 to 19. However, GE confirms that the system uses different factors as a function of dose between fluoro, high‐level fluoroscopy (HLF), and cine mode, as seen in this study (Table 5). CVs were also low for these two vendors: GE 0.58% and Philips 1.04%. Table 5 shows measured recursive filters for the average of three image series of several system settings, mentioned in Results and Discussion section. All results for all four vendors are given in Appendix A as a look‐up table. The GE C‐arm applies a higher recursive filter for a higher pulse rate and less recursive filtration with increasing dose.

**Table 4:** Accuracy and precision of the recursive filtration determination. The parameter settings are given by pulse rate (pps). Accuracy is the deviation of measured recursive filtration from the nominal (selected) recursive filtration, and the precision is given by the standard deviation (SD) and the coefficient of variation (CV). The calculation is based on 20 repeated image series. n/an indicates that the information is not available

C‐arm unit	Parameter settings (pps)	Nominal recursive filtration	Mean measured recursive filtration	Deviation from nominal (%)	SD	CV (%)
GE	8	Medium	2.68	n/a	0.0154	0.58
Philips	7.5	TNRT8	2.81	n/a	0.0292	1.04
Siemens	7.5	K = 4	4.03	1.08	0.0262	0.65
Ziehm	8	K = 4	4.01	0.31	0.0592	1.48

**Table 5:** Measured recursive filtration (average of three homogenous series) for different parameter settings. Some results from all four vendors are shown, making it possible to compare both within and between the vendors

Parameter settings Dose level: pps	Nominal recursive filtration	Measured recursive filtration K	Expected relative noise reduction	Relative noise reduction
GE				
HLF: 4 pps; 8 pps; 15 pps	Min	1.1;1.3;1.9	×;×;×	×;×;×
HLF: 4 pps; 8 pps; 15 pps	Low	1.2;1.7;3.1	×;×;×	×;×;×
HLF: 4 pps; 8 pps; 15 pps	Medium	1.6;2.7;5.3	×;×;×	×;×;×
HLF: 4 pps; 8 pps; 15 pps	High	1.7;2.9;5.8	×;×;×	×;×;×
Fluoro: 15 pps; Cine: 15 pps	Min	2.5;1.0	×;0.01	×;0.00
Fluoro: 15 pps; Cine: 15 pps	Low	4.0;1.1	×;0.09	×;0.19
Fluoro: 15 pps; Cine: 15 pps	Medium	6.7;1.3	×;0.23	×;0.35
Fluoro: 15 pps; Cine: 15 pps	High	7.6;2.4	×;0.48	×;0.50
Philips				
Fluoro: 4 pps;7.5 pps;15 pps	TNRT0	1.0;1.0;1.0	×;0.00;×	×;0.00;×
Fluoro: 7.5 pps	TNRT6	2.1	0.45	0.38
Fluoro: 4 pps;7.5 pps;15 pps	TNRT8	2.7;2.8;2.8	×;0.54;×	×;0.45;×
Fluoro: 7.5 pps	TNRT10	3.9	0.61	0.50
Fluoro: 4 pps(17.8 ms); 7.5 pps (32 ms); 15 pps (22.2 ms)	TNRT16	1.8;8.5;10.3	×;0.75;×	×;0.60;×
Fluoro:7.5 pps: low dose, normal dose, high dose	TNRT0	1.0;1.0;1.0	0.00;×;0.00	0.00;×;0.00
Fluoro:7.5 pps: low dose, normal dose, high dose	TNRT8	2.8;2.8;2.7	0.53;0.54;0,53	0.53;0.45;0,48
Fluoro:7.5 pps: low dose, normal dose, high dose	TNRT16	8.3;8.5;8.1	0.75;×:0,74	0.73; ×;0,65
Siemens				
Normal: 3 pps; 7.5pps; 15pps	K = OFF =1	1.0;1.0;1.0	×;0.00;×	×;0.00;×
Normal: 3 pps; 7.5pps; 15pps	K = 2	2.0;2.0;2.0	×;0.42;×	×;0.38;×
Normal: 3 pps;7.5 pps;15 pps	K = 4	3.7;4.0;4.0	×;0.62;×	×;0.57;×
Normal: 3 pps;7.5 pps;15 pps	K = 8	5.5;8.0;8.2	×;0.74;×	×0.68;×
Low: 7.5 pps; High:7.5 pps	K = 1	1.0;1.0	0.00;0.00;0.00	0.00;0.00;0,00
Low: 7.5 pps; High:7.5 pps	K = 4	4.0;4.0	0.62;0.62;0.62	0.57;0.57;0.57
Ziehm				
Normal: 4 pps; 8 pps; 12.5 pps	K = 1	1.0;1.0;1.0	×;0.00;×	×;0.00;×
Normal: 8 pps	K = 2	2.0	0.42	0.36
Normal: 4 pps; 8 pps; 12.5 pps	K = 4	3.9;4.0;4.0	×;0.62;×	×;0.52;×
Normal: 4 pps; 8 pps; 12.5 pps	K = 8	6.6;7.7;7.9	×;0.74;×	×;0.60;×
Normal: 8 pps	K = 16	13.0	0.80	0.65
Normal: 8 pps	ZAIP and K = 1	0.7 (4.3)*	‐0.58	0.00
Normal: 8 pps	ZAIP and K = 4	4.4 (26.8)*	0.64	0.11
Normal: 8 pps	ZAIP and K = 8	8.0 (48.9)*	0.74	0.16
Low: 7.5 pps; High:7.5 pps	K = 1	1.0;1.0	0.00;0.00	0.00;0.00
Low: 7.5 pps; High:7.5 pps	K = 4	3.9;4.0	0.62;0.62	0.39;0.44
Low: 7.5 pps; High:7.5 pps	K = 8	9.1;7.5	0.75;0.73	0.43;0.50

All results for all four vendors are given in Appendix A as a look‐up table. × denotes that no calculation is executed. *) effective filtration including ZAIP.

For the Philips C‐arm, the recursive filtration increased with increased number of TNRTs from 0 to 19, with a few exceptions, see Table 5. The preprogramed TNRTs are low, medium and high for the “reduce blur”, “none active”, and “reduce noise” buttons, respectively. Philips does not change the recursive filter due to dose, but similar to GE, Philips increases the recursive filtration for increasing pulse rate.

For Siemens, the measured recursive filtration was in accordance with the applied filtration, with the exception of a small deviation for low pulse rate (3 pps) and high filtration (K = 4 and 8).

The C‐arms from Ziehm display different images when the ZAIP is applied. The measured recursive filtration was in good agreement with the applied filtration when the ZAIP was off. Using ZAIP demands a correction of the calculated recursive filtration. The correction factor is linear with respect to the chosen recursive filtration. Similar to the Siemens C‐arm, the measured recursive filtration deviated from the selected one for low pulse rate (4) and high filtration factor (8 and 16).

### 4.2 Evaluation of noise reduction

The evaluation of noise reduction only considered the plateau given in Fig. 1, since it was demonstrated that the C‐arms omit the display of several of the first images in the series. Some C‐arms ignored showing approximately three images for 8 and 15 pps and K = 8. Another vendor ignored the first 800 ms of the series independent of K‐factor (3 images at 4 pps, 6 images at 8 pps, and 9 images at 12 pps), and some ignored as much as 3–4 s. Therefore, it became impossible to compare the noise from one frame to the corresponding frame in another series from the very beginning (time = 0). In addition, the C‐arms’ settings allow different recursive filtration for live images and last image hold images. Therefore, the last image was excluded from the analysis. From Fig. 1, it is seen that the noise reduction stabilizes after 3–30 images, depending on the K‐factor; hence, the noise reduction was determined for the plateau at n = 25.

The theoretical noise reduction estimated from the measured K‐factor and the noise reduction measured relative to series with K = 1 are presented in Table 5. For GE, the nominal recursive filtration is unknown. After analyzing the images, only one series turned out to have K = 1. For all other vendors, the noise reduction was determined for 7.5 pps. As expected, the image noise decreases with increasing recursive filtration. The measured noise reduction was smaller than the theoretical for all vendors except GE cine mode. The noise level was dependent on the recursive filtration and the image number in the series and independent of pulse rate and dose level.

The measured noise reductions for Philips TNRT 10, Siemens K = 4, and Ziehm K = 4 were 0.50, 0.57 and 0.52, respectively. Correspondingly, for Philips TNRT 16, Siemens, and Ziehm setting K = 8, the measured noise reductions were 0.60, 0.68 and 0.60, respectively.

## 5 Discussion

This study presents a novel method to determine the recursive filtration applied in a fluoroscopic series. Sometimes the recursive filtration is given in the system, but more often, it is unknown, as it is more common to use systems with adaptive recursive filtration. The user manual for the Philips Zenition indicates that standard image processing also uses adaptive temporal recursive noise reduction. High K‐factor gives blurred images when movement is present in the image. For systems that consider motion in the image, the noise suppression factor will automatically decrease as motion is detected. The level of temporal noise reduction adjusts according to the amount of movement in the region of interest (temporal recursive noise reduction) and to the applied pulse rate. Hence, the recursive filtration may not be constant across the image. Frequency‐dependent recursive filtration is another option. Detection of poor contrast for high‐resolution objects may increase the recursive filtration. In addition, for Siemens, it is possible to change the applied K‐factor and to choose whether the system should consider movement. For Ziehm, it is possible to select Auto to activate recursive filtration, and ZAIP gives improved images with moving objects by regulating filters in real time. Using ZAIP and a K‐factor of one, the corrected measured K‐factor was 0.7, which should not be possible. The ZAIP reduces the effect of lag due to recursive filtration; hence, it is not intended to use ZAIP when recursive filtration is off. Instead, it might lead to an increase in noise; however, the evaluation of lag due to recursive filtration and assessment of image quality is beyond the scope of this study. Regardless of whether the recursive filtration is adaptive, the applied filtration has great impact on the noise in the displayed image. Therefore, it is important to know the applied recursive filtration in the actual image acquired for noise evaluation as part of quality control, such as SNR and contrast‐to‐noise ratio (CNR) measurements. The applied recursive filtration can be measured using the method presented in this study.

For simplicity, recursive filtration is interpreted as an average of a certain number of images, but as seen from Table 5, the recursive filtration may not be an integer. The reason is that the recursive filtration is a weighting of the images where a portion of the signal from previous frames is added into the current frame. The recursive filtration and corresponding noise reduction was measured for C‐arms from four different vendors. The method to determine the recursive filtration had good precision (range 0.31%–1.08%) and accuracy given by the CV range 0.58% to 1.48%. Kotre and Guibelalde have published a study were a time constant is measured as function of applied K‐factor in an X‐ray system using image intensifiers.^16^ The quantum noise is expected to reduce approximately as the reciprocal of the square root of the temporal averaging time constant. The noise reductions calculated based on their relative temporal averaging constants are 0.42, 0.60, 0.7, and 0.79 for K‐factors of 2, 4, 8, and 16, in excellent agreement with the theoretical noise reduction (based on K‐factor) in Table 5 for K = 2 and 4 and slightly lower for K = 8 and 16. Nevertheless, they determine the temporal averaging constant by using the correlation of the variance between the initial frame and successive frames.^4^ The correlation method is not applicable for the flat panel detector C‐arms investigated in this study since they omit several frames in the beginning of a series. However, our proposed method is applicable for both image intensifier and flat panel detectors, given that it is possible to save the image series.

When the user selects low pps and high recursive filtration, the system automatically reduces the applied recursive filtration. This can be seen from Table 5 for Philips, Siemens, and Ziehm. For GE, it is also seen that the K‐factor increases with increasing pps, even though the user is not able to choose the K‐factor setting. Table 5 gives the measured K‐factors for most of the selectable recursive filtration settings in the systems. As an example for comparison, it is seen that the GE settings for the General HD protocol, 15 pps fluoro, low recursive filtration is equal to Philips TNRT 10, Siemens K = 4 and Ziehm setting K = 4. The highest applied recursive filtration for the GE system using protocol General HD was 7.6 for fluoro, 15 pps. This is similar to Philips TNRT 16 and the Siemens and Ziehm setting K = 8. Figure 1 demonstrates that for β less than 0.125 (K higher than 8), more than 15 images are required to stabilize the noise.

The results clearly show that application of recursive filtration affects the noise in the image and has to be taken into consideration when image quality for a system is evaluated. With the exception of the C‐arm from GE cine mode, none of the C‐arms achieves the theoretical noise reduction. The theory considers stochastic noise only. However, the measured noise reduction based on two series also contains noise that does not become reduced with recursive filtration such as fixed pattern (FP) noise. The FP noise, given as the deviation between the expected noise reduction and measured noise reduction using two image series, seems to increase for increasing K‐factor for the C‐arms from Philips and Ziehm (Table 5). This is also in accordance with Ref. 16. Hence, the measured noise reduction will be smaller than the theoretical noise reduction. It is not obvious why the GE C‐arm performs better than expected from the measured K‐factor, but it might result from some postprocessing that is confidential. This may also apply to Ziehm ZAIP, which reduces lag (results not included in this study) but probably does not reduce noise to the extent that is expected, as seen in Table 5.

The achieved noise reduction due to recursive filtration comes at a cost of increased lag in the images. Hence, the lag should also be quantified, which will be the focus of a future study.

## 6 Conclusion

Noise evaluation is a common task in quality control. The noise level strongly depends on the applied recursive filtration and the fluoroscopic exposure time until the standard deviation stabilizes. Therefore, it is important to take the recursive filtration into account when a phantom is imaged for quality assurance purposes. This study presents a novel method to determine the actual applied recursive filtration in a fluoroscopic series and makes it possible to compare recursive settings between different vendors.

## Appendix 1

**Table A1:** Measured recursive filtration (average of three homogenous series) for different parameter settings. All results for all four vendors are included which makes it possible to compare within a vendor and between the vendors

Parameter settings Dose level: pps: FOV**	Nominal recursive filtration	Measured recursive filtration K	Expected relative noise reduction	Relative noise reduction
GE				
HLF: 4 pps; 8 pps; 15 pps	Min	1.1;1.3;1.9	×;×;×	×;×;×
HLF: 4 pps; 8 pps; 15 pps	Low	1.2;1.7;3.1	×;×;×	×;×;×
HLF: 4 pps; 8 pps; 15 pps	Medium	1.6;2.7;5.3	×;×;×	×;×;×
HLF: 4 pps; 8 pps; 15 pps	High	1.7;2.9;5.8	×;×;×	×;×;×
Fluoro: 15 pps; Cine: 15 pps	Min	2.5;1.0	×;0.01	×;0.00
Fluoro: 15 pps; Cine: 15 pps	Low	4.0;1.1	×;0.09	×;0.19
Fluoro: 15 pps; Cine: 15 pps	Medium	6.7;1.3	×;0.23	×;0.35
Fluoro: 15 pps; Cine: 15 pps	High	7.6;2.4	×;0.48	×;0.50
Philips				
Fluoro: 4 pps;7.5 pps;15 pps	TNRT0	1.0;1.0;1.0	×;0.00;×	×;0.00;×
Fluoro: 7.5 pps	TNRT1	1.2	0.12	0.11
Fluoro: 7.5 pps	TNRT2	1.4	0.24	0.21
Fluoro: 7.5 pps	TNRT3	1.7	0.35	0.30
Fluoro: 7.5 pps	TNRT4	1.7	0.36	0.31
Fluoro: 7.5 pps	TNRT5	2.2	0.45	0.38
Fluoro: 7.5 pps	TNRT6	2.1	0.45	0.38
Fluoro: 7.5 pps	TNRT7	2.8	0.53	0.45
Fluoro: 4 pps;7.5 pps;15 pps	TNRT8	2.7;2.8;2.8	×;0.54;×	×;0.45;×
Fluoro: 7.5 pps	TNRT9	3.6	0.60	0.49
Fluoro: 7.5 pps	TNRT10	3.9	0.61	0.50
Fluoro: 7.5 pps	TNRT11	4.7	0.66	0.53
Fluoro: 7.5 pps	TNRT12	5.2	0.67	0.54
Fluoro: 7.5 pps	TNRT13	5.7	0.69	0.55
Fluoro: 7.5 pps	TNRT14	6.7	0.72	0.57
Fluoro: 7.5 pps	TNRT15	7.0	0.72	0.58
Fluoro: 4 pps(17.8 ms);7.5 pps (32 ms);15 pps(22.2 ms)	TNRT16	1.8;8.5;10.3	×;0.75;×	×;0.60;×
Fluoro: 7.5 pps	TNRT17	7.2	0.73	0.59
Fluoro: 7.5 pps	TNRT18	9.1	0.76	0.61
Fluoro: 7.5 pps	TNRT19	11.3	0.78	0.62
Fluoro:7.5 pps:low dose, normal dose, high dose	TNRT0	1.0;1.0;1.0	0.00;×;0.00	0.00;×;0.00
Fluoro:7.5 pps:low dose, normal dose, high dose	TNRT8	2.8;2.8;2.7	0.53;0.54;0.53	0.53;0.45;0.48
Fluoro:7.5 pps:low dose, normal dose, high dose	TNRT16	8.3;8.5;8.1	0.75;×;0.74	0.73;×;0.65
Siemens				
Normal: 3 pps;7.5 pps;15 pps	K = OFF =1	1.0;1.0;1.0	×;0.00;×	×;0.00;×
Normal: 3 pps;7.5 pps;15 pps	K = 2	2.0;2.0;2.0	×;0.42;×	×;0.38;×
Normal: 3 pps;7.5 pps;15 pps	K = 4,	3.7;4.0;4.0	×;0.62;×	×;0.57;×
Normal: 3 pps;7.5 pps;15 pps	K = 8	5.5;8.0;8.2	×;0.74;×	×;0.68;×
Low: 7.5 pps; High:7.5 pps	K = 1	1.0;1.0	0.00;0.00;0.00	0.00;0.00;0,00
Low: 7.5 pps; High:7.5 pps	K = 4	4.0;4.0	0.62;0.62;0.62	0.57;0.57;0.57
Normal: 7.5 pps: FOV2;FOV3	K = 1	1.0;1.0	×;×	×;×
Normal: 7.5 pps: FOV2;FOV3	K = 4	4.0;4.0	×;×	×;×
Normal: 7.5 pps	K = 4 (weight factor 0.7)	2.8	×	×
Normal: 7.5 pps	K = 4 (weight factor 1.4)	5.6	×	×
Ziehm				
Normal: 4 pps; 8 pps; 12.5 pps	K = 1	1.0;1.0;1.0	×;0.00;×	×;0.00;×
Normal: 8 pps	K = 2	2.0	0.42	0.36
Normal: 8 pps	K = 3	3.0	0.55	0.47
Normal: 4 pps; 8 pps; 12.5 pps	K = 4	3.9;4.0;4.0	×;0.62;×	×;0.52;×
Normal: 8 pps	K = 5	5.0	0.67	0.56
Normal: 8 pps	K = 6	6.0	0.70	0.58
Normal: 8 pps	K = 7	7.0	0.72	0.59
Normal: 4 pps; 8 pps; 12.5 pps	K = 8	6.6;7.7;7.9	×;0.74;×	×;0.60;×
Normal: 8 pps	K = 9	8.8	0.75	0.61
Normal: 8 pps	K = 10	9.4	0.76	0.62
Normal: 8 pps	K = 11	10.0	0.77	0.63
Normal: 8 pps	K = 12	10.9	0.78	0.63
Normal: 8 pps	K = 13	11.7	0.79	0.64
Normal: 8 pps	K = 14	12.3	0.79	0.64
Normal: 8 pps	K = 15	13.0	0.79	0.65
Normal: 8 pps	K = 16	13.0	0.80	0.65
Normal: 8 pps	ZAIP and K = 1	0.7 (4.3)*	‐0.58	0.00
Normal: 8 pps	ZAIP and K = 2	2.1 (12.6)*	0.44	0.06
Normal: 8 pps	ZAIP and K = 3	3.3(20.0)*	0.58	0.08
Normal: 8 pps	ZAIP and K = 4	4.4(26.8)*	0.64	0.11
Normal: 8 pps	ZAIP and K = 5	5.6(33.8)*	0.69	0.12
Normal: 8 pps	ZAIP and K = 6	6.4(39.2)*	0.71	0.15
Normal: 8 pps	ZAIP and K = 7	7.2(44.0)*	0.73	0.16
Normal: 8 pps	ZAIP and K = 8	8.0(48.9)*	0.74	0.16
Normal: 8 pps	ZAIP and K = 9	8.8(53.8)*	0.75	0.20
Normal: 8 pps	ZAIP and K = 10	9.2(56.3)*	0.76	0.21
Normal: 8 pps	ZAIP and K = 11	9.6(58.6)*	0.77	0.22
Normal: 8 pps	ZAIP and K = 12	10.2(61.9)*	0.77	0.21
Normal: 8 pps	ZAIP and K = 13	11.1(67.7)*	0.78	0.22
Normal: 8 pps	ZAIP and K = 14	11.0(67.0)*	0.78	0.23
Normal: 8 pps	ZAIP and K = 15	12.3(74.7)*	0.79	0.23
Normal: 8 pps	ZAIP and K = 16	11.7(71.4)*	0.79	0.23
Low: 7.5 pps; High:7.5 pps	K = 1	1.0;1.0	0.00;0.00	0.00;0.00
Low: 7.5 pps; High:7.5 pps	K = 4	3.9;4.0	0.62;0.62	0.39;0.44
Low: 7.5 pps; High:7.5 pps	K = 8	9.1;7.5	0.75;0.73	0.43;0.50

× denotes that no calculation is executed. ^*)^effective filtration including ZAIP. **) Only specified when FOV differ from default normal mode.
